# New Insights into the Clinical Characterization of *SDHAF2*-related Familial Paraganglioma Syndrome

**DOI:** 10.1210/clinem/dgaf149

**Published:** 2025-03-13

**Authors:** Antia Fernandez-Pombo, Zulema Nogareda-Seoane, Jose Manuel Cameselle-Teijeiro, Ana Ecenarro-Montiel, Cecilia Vieira-Leite, Gemma Rodriguez-Carnero, Noelia Otero-Mato, Virginia Pubul-Nuñez, Marcos Pazos-Couselo, Lourdes Loidi, Jose Manuel Cabezas-Agricola

**Affiliations:** Division of Endocrinology and Nutrition, University Clinical Hospital of Santiago de Compostela, 15706 Santiago de Compostela, Spain; Unidad de Enfermedades Tiroideas e Metabólicas (UETeM)-Molecular Pathology Group, Department of Psychiatry, Radiology, Public Health, Nursing and Medicine, Center for Research in Molecular Medicine and Chronic Diseases (CIMUS), University of Santiago de Compostela, 15782 Santiago de Compostela, Spain; Epigenomics in Endocrinology and Nutrition Group, Epigenomics Unit, Instituto de Investigacion Sanitaria de Santiago de Compostela (IDIS), University Clinical Hospital of Santiago de Compostela, 15706 Santiago de Compostela, Spain; Division of Nuclear Medicine, University Clinical Hospital of Santiago de Compostela, 15706 Santiago de Compostela, Spain; Department of Pathology, University Clinical Hospital of Santiago de Compostela, Health Research Institute of Santiago de Compostela (IDIS), University of Santiago de Compostela (USC), 15706 Santiago de Compostela, Spain; Division of Radiology, University Clinical Hospital of Santiago de Compostela, 15706 Santiago de Compostela, Spain; Division of Radiology, University Clinical Hospital of Santiago de Compostela, 15706 Santiago de Compostela, Spain; Division of Endocrinology and Nutrition, University Clinical Hospital of Santiago de Compostela, 15706 Santiago de Compostela, Spain; Epigenomics in Endocrinology and Nutrition Group, Epigenomics Unit, Instituto de Investigacion Sanitaria de Santiago de Compostela (IDIS), University Clinical Hospital of Santiago de Compostela, 15706 Santiago de Compostela, Spain; Division of Endocrinology and Nutrition, University Clinical Hospital of Santiago de Compostela, 15706 Santiago de Compostela, Spain; Division of Nuclear Medicine, University Clinical Hospital of Santiago de Compostela, 15706 Santiago de Compostela, Spain; Department of Psychiatry, Radiology, Public Health, Nursing and Medicine, University of Santiago de Compostela, 15705 Santiago de Compostela, Spain; Division of Molecular Medicine, Galician Public Foundation for Genomic Medicine (SERGAS-Xunta de Galicia), 15706 Santiago de Compostela, Spain; Division of Endocrinology and Nutrition, University Clinical Hospital of Santiago de Compostela, 15706 Santiago de Compostela, Spain

**Keywords:** *SDH*, *SDHAF2*, paraganglioma, pheochromocytoma, PET, GAPP

## Abstract

**Background:**

The clinical characterization of *SDHAF2*-related familial paraganglioma syndrome remains elusive. The aim of this study is to contribute to the knowledge of this syndrome with valuable new information.

**Methods:**

A total of 56 individuals with the p.(Gly78Arg) variant in the *SDHAF2* gene were prospectively evaluated. Of the 33 subjects who developed paragangliomas (PGLs)/pheochromocytomas (PCs) throughout follow-up, clinical, biochemical, and imaging data were collected. [68Ga]Ga-DOTA-TOC and [18F]DOPA positron emission tomography/computed tomography (PET/CT) scans were carried out on a subset of 22 patients with PGLs/PCs to compare their accuracy; surgical specimens (n = 13) were microscopically evaluated to elucidate their potential malignant behavior.

**Results:**

Of the 33 patients (58.9%) with *SDHAF2*-related tumors, 17 (51.5%) were women, with a mean age at diagnosis of 38.6 ± 17.2 years. Tumor development was found to be inherited paternally in all subjects. All the patients evaluated except 1 showed head and neck PGLs. Eleven patients (33.3%) showed mediastinal and abdominal extra-adrenal PGLs and 2 patients presented PCs. Multifocality was observed in 26 subjects (78.8%). Sixteen patients (48.5%) were asymptomatic at diagnosis. Only 4 patients with PGLs/PCs showed normetanephrine or 3-methoxytyramine secretion. Metastatic disease was observed in 2 patients (6.1%). Grading System for Adrenal Pheochromocytoma and Paraganglioma score was ≥3 in 84.6% of tumors and Pheochromocytoma of the Adrenal Gland Scaled Score was ≥4 in 69.2%. [68Ga]Ga-DOTA-TOC PET/CT showed a greater detection rate (95.7%) of multifocal PGLs and metastatic lesions than [18F]DOPA PET/CT (79.3%), as well as higher mean maximum standardized uptake value.

**Conclusion:**

The current study offers new insights into the phenotypic characterization of *SDHAF2*-related paraganglioma syndrome including the development of extra-cervical PGLs and metastatic transformation.

Paragangliomas (PGLs) and pheochromocytomas (PCs) are rare neural crest-derived tumors composed of chromaffin tissue, located at the extra-adrenal level or affecting the adrenal medulla, respectively. Their global annual incidence accounts for 2 to 8 cases/million inhabitants (1-3).

Around 70% to 80% of patients with these tumors are affected by germline or somatic pathogenic variants in more than 20 susceptibility genes (4-9). Thus, data from The Cancer Genome Atlas have classified PGLs/PCs into 3 clinically relevant clusters based on these specific gene variants, with succinate dehydrogenase (SDH) subunit variants being included within cluster 1 or pseudohypoxia group, involved in metabolic or oxygen-sensing pathways (4-6, 10). The importance of this classification is that it translates into clinical, biochemical, and imaging signatures, which may guide follow-up and therapy.

Germline pathogenic variants in genes encoding SDH subunits (*SDHA*, *SDHB*, *SDHC*, *SDHD*; collectively named SDHx) are considered to be a frequent cause of PGLs and PCs, accounting for ∼20% of all cases (11, 12). In addition, the SDH complex assembly factor 2 (SDHAF2) is essential for the correct flavination of SDHA and the function of the entire SDH complex. The loss of this protein leads to a reduction of the stability of the complex and, therefore, to a decrease in the amount of all subunits (13). The first pathogenic variant identified in the *SDHAF2* gene (p.(Gly78Arg)) was described in 2009 in a Dutch family showing head and neck PGLs (13-16). Since then, to the best of our knowledge, only 1 other family with the same variant has been described in Spain, in addition to single cases in the United States, Ireland, Italy, Switzerland, United Kingdom, and the Netherlands (17-23). Another isolated case with the p.N146K variant and metastatic PC has also been reported (24).

Current knowledge about *SDHAF2*-related familial paraganglioma syndrome, through these 2 families and single cases, is that it only associates with parasympathetic head and neck PGLs, the presence of PCs is rare (with only 2 cases described in the literature) and only 2 isolated cases with metastatic disease have been reported. *SDHAF2* (11q13), like *SDHD* (11q23), also shows a notable parental origin effect, in which tumor development depends almost entirely on paternal transmission of the mutant allele due to different complex mechanisms (13-26).

Taking into account the rarity of this specific syndrome and that current knowledge is based exclusively on the study of individual cases or reports limited to small samples, the aim of this study was to determine the clinical, biochemical, and imaging profile of *SDHAF2*-related familial paraganglioma syndrome through a large cohort of individuals with the c.232G > A p.(Gly78Arg) variant, in an effort to contribute to the understanding of this disorder.

## Subjects and Methods

### Study Population and Design

Between 2001 and 2023, a total of 90 individuals from 7 index cases belonging to 7 families with the pathogenic variant p.(Gly78Arg) in the *SDHAF2* gene were evaluated at the Endocrinology and Nutrition Department of the University Clinical Hospital of Santiago, Spain. This department attends patients from the health care area of Santiago de Compostela and Barbanza (Galicia, Spain), with a total population of ∼450 000 patients (27). After molecular screening of family members, from these 90 individuals, a total of 56 subjects were found to be carriers of this variant and 33 developed PGLs/PCs.

These patients were evaluated by the same senior endocrine physician (J.M.C.-A.), with the support of a multidisciplinary endocrine tumor board constituted by specialists in endocrinology, endocrine surgery, oncology, radiology, nuclear medicine, and pathology. All patients carrying the previously mentioned variant in the *SDHAF2* gene underwent a biochemical study with urinary catecholamines and metanephrines and anatomical imaging study with diagnostic magnetic resonance imaging (MRI) or computed tomography (CT). In addition, both [18F]DOPA and [68Ga]Ga-DOTA-TOC positron emission tomography (PET)/CT scans were carried out on 22 patients with PGLs/PCs to compare their accuracy. The clinical and demographic data of the patients were obtained from their electronic medical records. Multifocality was defined as the presence of tumors in multiple sites. Malignancy was defined by the presence of tumor deposits in regions in which chromaffin cells are physiologically absent (ie, lymph nodes, lungs, bone). The course of treatment in each of the patients was decided after exhaustive evaluation by the multidisciplinary board. The surgical specimens obtained were evaluated.

All procedures performed in this study were carried out in accordance with the ethical standards of the Ethical Review Panel of the Red Gallega de Comités de Ética de la Investigación (registration code 2022/025) in accordance with the 2013 Declaration of Helsinki and its subsequent modifications or comparable ethical standards. All subjects gave their informed consent to participate in the study and for the publication of their clinical, biochemical, and molecular data.

### Biochemical Study

The study of 24-hour urine catecholamines and metanephrines (adrenaline, norepinephrine, metanephrine, normetanephrine, dopamine, and 3-methoxytyramine) was carried out in all patients, using containers with 10 mL of 6M hydrochloric acid. Patients were urged not to consume food or medications that could interfere with the results on the day before and the day of urine collection. For this analysis, liquid chromatography coupled with mass spectrometry was used, with standardized methods and appropriate quality control and quality assurance procedures per laboratory protocol. To verify adequate and complete urine collection, urine creatinine levels were also measured.

### Molecular Study

The search for variants was performed by next-generation sequencing (Illumina NextSeq 500) of the entire coding region and the flanking intronic regions of the *EGLN1, EPAS1, FH, KIF1B, MAX, MEN1, NF1, RET, SDHA, SDHAF2, SDHB, SDHC, SDHD, TMEM127,* and *VHL* genes for all the index patients evaluated, followed by supplementary assays when needed for the interpretation of the results. Data analysis was carried out using the following software: fastp v0.20.1, bwa 0.7.17-r1188, GATK v3.8-0, GATK v4.1.8.0, Pindel 0.2.5b9, Picard 2.23.1, mosdepth 0.2.9, bedtools v2.29.2, samtools 1.10, ExomeDepth 1.1.15, and ANNOVAR 2020Jun7. The interpretation and classification of variants was carried out according to the recommendations of the American College of Medical Genetics and Genomics (28). The coding region of the analyzed genes presents an analysis coverage of 100%, except for *NF1*, the coverage of which is >95%. Analysis coverage calculation was performed on the target region, exons, and flanking intronic regions up to 10 bp with read depth of at least 30×. The average read depth of the genes was 277×. The carrier status of family members (symptomatic or asymptomatic) was performed by PCR and direct Sanger sequencing of *SDHAF2* exon 2.

### Imaging Study With PET/CT

Both [18F]DOPA and [68Ga]Ga-DOTA-TOC PET/CT scans (Philips Vereos) were carried out prospectively on a total of 22 patients with a maximum period of time between both imaging techniques of 6 months. During this period, no therapy or intervention was performed.

#### [18F]DOPA PET/CT

The study of the intracellular transport and decarboxylation of the amino acid dihydroxyphenylalanine was carried out by PET after the IV injection of an activity of between 2 and 4 MBq/kg of [18F]DOPA. Delayed images from the top of the skull to mid-thigh were obtained at 60 minutes after injection, with acquisition in 3-dimensional mode and attenuation correction using low-dose CT imaging without contrast. Lesion quantification was performed by measuring the maximum standardized uptake value (SUV_max_), which is expressed in grams per milliliter. To avoid interaction with amino acids from food, the radiopharmaceutical was administered after a minimum of 4 hours’ fasting, without limiting water intake.

#### [68Ga]Ga-DOTA-TOC PET/CT

The study of somatostatin receptors was carried out by PET after the IV injection of an activity of between 100 and 200 MBq of [68Ga]Ga-DOTA-TOC, obtaining images from the top of the skull to mid-thigh at 50 minutes after injection, with acquisition in 3-dimensional mode and attenuation correction by low-dose CT without contrast. Lesion quantification was performed by measuring SUV_max_, which is expressed in grams per milliliter.

### Histopathological Study

The microscopic features of a subset (n = 13) of PGLs (representative of the head and neck, extra-cervical region, and metastatic PGLs) and PCs from patients for whom specimen samples were available after surgery were exhaustively evaluated. Tumor tissue samples were fixed in 10% neutral buffered formalin, embedded in paraffin by standard procedure, sectioned at 4-μm thickness, and mounted on silane-coated glass slides. Consecutive tissue sections were stained with hematoxylin-eosin for histological diagnosis including both the Pheochromocytoma of the Adrenal Gland Scaled Score (PASS) (29) and the Grading System for Adrenal Pheochromocytoma and Paraganglioma (GAPP) score (30) to elucidate the potential malignant behavior of the tumors. For this purpose, additional immunostainings for Ki-67, S100, and SDHB were carried out. Immunohistochemistry was performed using a peroxidase-conjugated labeled dextran polymer (EnVision FLEX/HRP; Dako, Glostrup, Denmark), with 3,3′-diaminobenzidine as the chromogen (GC80611-2; Dako) in an automatic immunostainer (Autostainer Link 48; Agilent, Santa Clara, CA). The primary antibodies (clone, concentration, antigenic recovery treatment, and manufacturer) were as follows: Ki-67 (MIB1, ready to use, pH 6; Dako), S100 (polyclonal, ready to use, pH 9, Dako), and SDHB (ZR339, 1/200, pH 6, Gennova, Seville, Spain). Primary antibodies were substituted for nonimmune mouse and rabbit serum samples as negative control samples.

### Statistical Analysis

Data were expressed as n (%) and mean ± standard deviation. The hypothesis of a normal distribution was verified by the Shapiro-Wilk test. The detection rate of functional imaging (%) was assessed on a per-patient basis using the McNemar test. The paired *t*-test was used to determine the differences between the mean SUV_max_ of the lesions according to both [18F]DOPA and [68Ga]Ga-DOTA-TOC PET/CT. The statistical significance level was set at *P* < .05. Statistical analysis was performed using the SPSS 22.0 program (Chicago, IL, USA).

## Results

### Demographic Data and Inheritance

Of the total of 90 individuals evaluated from 7 index cases belonging to 7 families with the p.(Gly78Arg) variant in the *SDHAF2* gene, 56 (67.5%) harbored this pathogenic variant. Of these, 33 developed PGLs/PCs between 2001 and 2023. Therefore, the global penetrance of this specific genetic change (58.9%) was shown to be high. If only the family members that inherited the pathogenic variant from their father are taken into account, the penetrance rises to 87.5%. The remaining 23 (41.1%) subjects were either considered to be merely carriers of the disease-associated variant or they had not yet developed the disease because of their young age.

**Figure 1.**
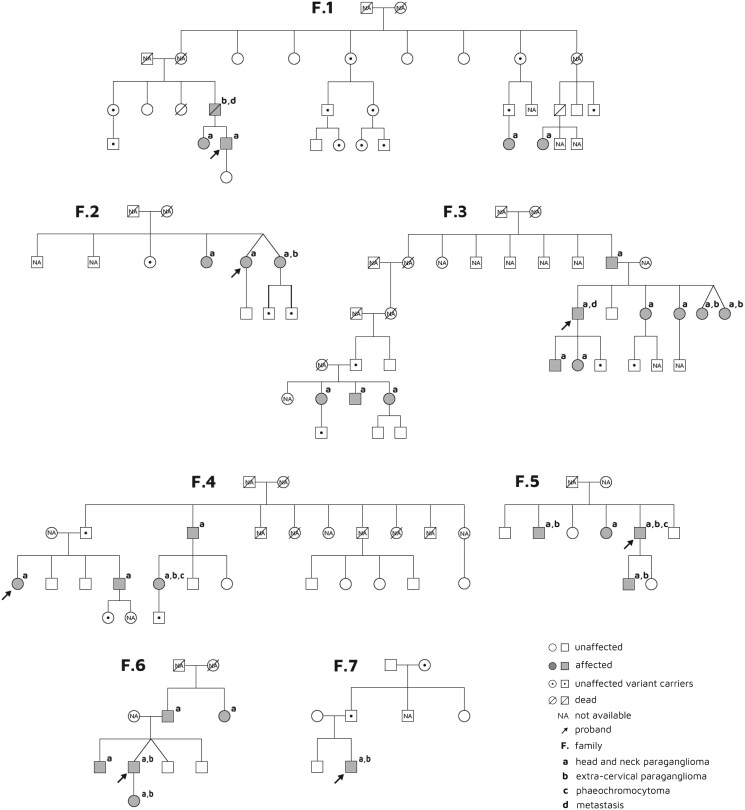
Pedigree chart of the seven families with *SDHAF2*-related familial paraganglioma syndrome.

Of the 33 patients that developed the *SDHAF2*-related familial paraganglioma syndrome, 18 (54.5%) were women, with a mean age of 48.9 ± 18.3 years. Mean age at diagnosis, considering the detection of the first PGLs/PCs via MRI or CT scan, was 38.6 ± 17.2 (range, 12.8-78.7) years. Median follow-up from diagnosis was 10.0 (1.1-38.0) years. Tumor development was found to be inherited paternally in all cases, following an autosomal dominant inheritance pattern. Figure 1 shows the pedigree chart of the affected families.

### Clinical and Biochemical Characterization

Table 1 summarizes the main clinical data of the 33 patients at the time of diagnosis of PGLs/PCs and follow-up. A high degree of multifocality was observed, with 26 (78.8%) patients presenting multiple PGLs in different locations. All the patients evaluated except 1 showed head and neck PGLs, mainly at the carotid bifurcation area (24 [72.7%] patients), followed by the jugular and jugulotympanic regions (15 [45.4%] and 10 [30.3%] patients, respectively). Four (12.1%) patients showed head and neck PGLs in other locations, such as the glomus vagal and the common carotid artery areas. Interestingly, a total of 11 patients (33.3%) presented extracervical PGLs and 2 patients PCs. More specifically, 9 patients showed mediastinal PGLs, particularly in the interventricular sulcus and the epicardial, retrocardiac, paraaortic, aortic arch, and paratracheal regions; 3 patients manifested abdominal extra-adrenal PGLs. In fact, the only patient without head and neck PGLs was diagnosed with a 14.3-cm nonsecreting retrocardiac PGL at the age of 59 years after consulting for dyspnea and orthopnea. The largest mean size of PGLs/PCs evaluated through MRI or CT scan was 3.9 ± 2.5 cm, with a tumor size range of 0.7 to 14.3 cm.

**Table 1.** Clinical and biochemical data at first visit and follow-up of patients with SDHAF2-related familial paraganglioma syndrome

Clinical data of PGLs/PCs at presentation (n = 33)	
Location of PGLs/PCs	
Head and neck [n patients (%)]	32 (97.0)
Carotid bifurcation area [n patients (%)]	24 (72.7)
Jugular area [n patients (%)]	15 (45.4)
Jugulotympanic area [n patients (%)]	10 (30.3)
Other areas [n patients (%)]	4 (12.1)
Extracervical [n patients (%)]	11 (33.3)
Mediastinal [n patients (%)]	9 (27.3)
Abdominal extra-adrenal [n patients (%)]	3 (9.1)
Adrenal [n patients (%)]	2 (6.1)
Size of the PGL/PC (cm)	3.9 ± 2.5
Multifocality [n patients (%)]	26 (78.8)
Bilateral [n patients (%)]	25 (75.7)
Clinical presentation	
Asymptomatic [n patients (%)]	16 (48.5)
Tumour appearance [n patients (%)]	10 (30.3)
Otological symptoms [n patients (%)]	5 (15.1)
Others [n patients (%)]	3 (9.1)
Catecholamine/metanephrine secretion [n patients (%)]*^a^*	4 (12.1)
**Clinical data at follow-up and treatment received (n** = **33)**	
Treatment	
Surgery [n patients (%)]	28 (72.7)
Radiosurgery [n patients (%)]	3 (9.1)
Radiotherapy [n patients (%)]	8 (24.2)
Tyrosine kinase inhibitor [n patients (%)]	1 (3.0)
Surveillance [n patients (%)]	2 (6.1)
Complications [n patients (%)]	15 (45.4)
Malignant transformation [n patients (%)]	2 (6.1)
Development of other tumors [n patients (%)]	5 (15.1)
Death [n patients (%)]	1 (3.0)

Data are expressed as n (%) patients or mean ± SD.

Abbreviations: PC, pheochromocytoma; PGL, paraganglioma; SDHAF2, SDH complex assembly factor 2.

^*a*^Greater than 3-fold above the upper limit of normality.

Regarding the clinical presentation of the disease, 16 (48.5%) patients were asymptomatic and PGLs were found through imaging study following the detection of the pathogenic variant in the cascade screening. As for symptomatic patients, the most common symptoms were the appearance of a palpable tumor and the presence of otological symptoms such as tinnitus and hearing loss. The 2 patients with PCs presented with hypertensive crisis in addition to symptoms from the classic triad such as headache, hyperhidrosis, or tachycardia.

On the other hand, most of the patients exhibited nonsecreting PGLs. However, the secretion of normetanephrine was observed in the 2 patients with PCs and in 1 patient with abdominal PGLs. An increased secretion of 3-methoxytyramine was also observed in 1 patient with head and neck PGLs and dopamine levels within the normal range.

Table 2 synthesizes the available clinical data regarding the families and individual cases previously described in the literature with the *SDHAF2*-related familial paraganglioma syndrome (13, 14, 16-23 + Denes2015), along with the new Spanish cohort presented here. An overall estimation of the prevalence of these clinical features can be seen in the table.

**Table 2.** Summary of the main clinical features of the families and individual cases with SDHAF2-related familial paraganglioma syndrome reported to date and estimation of the overall prevalence

	Netherlands 1982-2009 (13, 14)	Netherlands 2011 (16)	Netherlands 2024 (22)	Spain 2010 (17)	Italy 2012 (19)	Ireland 2014 (21)	United Kingdom 2015 (23)	Saudi Arabia 2019 (24)	United States 2019 (18)	Switzerland 2020 (20)	Spain 2024	Overall
Variant	c.232G > A	c.232G > A	c.232G > A	c.232G > A	c.232 G > C c.357_358insT	c.12C > T	c.-52T > C	c.438C > A	c.347G > A	c.232G > A	c.232G > A	—
Individuals tested (n)	89	57	4	6	79 patients with PGLs*^a^*	31 patients with PGLs/PCs*^a^*	39 patients with PGLs/PCs*^a^*	101 patients with PGLs/PCs*^a^*	10	1	90	507
Carriers (n)	45	23	4	4	2	2	1	1	6	1	56	145
Patients with PGLs/PCs (n)	33	11	4	4	2	2	1	1	1	1	33	93
Penetrance (%)*^b^*	85%	75%	—	100%	—	—	—	—	33%	—	88%	76%
Inheritance	AD Paternal transmission	AD Paternal transmission	AD Paternal transmission	AD Paternal transmission	Negative family history	Negative family history	Negative family history	Negative family history	AD Paternal transmission	N/A	AD Paternal transmission	AD Paternal transmission
Gender*^c^*	77% male 23% female	55% female 45% male	75% male 25% female	75% female 25% male	N/A	100% male	Male	N/A	Female	Male	55% female 45% male	58% male 42% female
Average age at onset (y)	74	33	14	31	N/A	56	84	N/A	30	15	39	42
Metastases	Not described	No	Yes (n = 1, lungs, spinal bone, pancreas*^d^*)	Not described	Not described	No	Not described	Yes	Not described	No	Yes (n = 2, lungs, bone)	4% (mainly lungs and bone)
Multifocality	Yes (50%)	Yes (91%)	Yes (100%)	Yes (50%)	No	No	No	No	No	Yes (100%)	Yes (79%)	66%
Location of PGLs/PCs	Head and neck (JTT > CT > VT) Mediastinal PGL (n = 1)	Head and neck (CT > VT > JTT)	Head and neck (CT, VT JTT)	Head and neck (CT > VT and JTT > intrathyroid)	Head and neck PGL (CT, JTT)	Adrenal PCs	Head and neck	Adrenal PC	Head and neck (CT)	Head and neck (CT, JTT)	Head and neck (CT > JTT > VT) Extra-cervical PGLs (n = 11) Adrenal PCs (n = 2)	Mainly head and neck PGLs (96%) Extra-cervical PGLs (13%) Adrenal PCs (5%)
Average tumour size	N/A	N/A	3.0 cm	N/A	N/A	4.5 cm	N/A	N/A	4.5 cm	2.5 cm	3.9 cm	3.7 cm
Biochemical pattern	N/A	N/A	Non-secreting (free 3MT slightly increased)	N/A	Non-secreting	Secreting PCs (MN, NMN)	N/A	N/A	Non-secreting	Non-secreting	Mainly non-secreting NMN 3MT	Mainly non-secreting (86%)*^e^* NMN (9%)*^e^* MN (2%)*^e^* 3MT (2%)*^e^*
Other tumors	N/A	N/A	N/A	N/A	N/A	No	Somatotroph PA	N/A	Papillary thyroid carcinoma, follicular adenoma	N/A	Oesophageal, cervical and endometrial cancer Ovarian endometrioma Lipomas	—

Abbreviations: 3MT, 3-methoxytyramine; CT, carotid tumor; JTT, jugular/jugulotympanic tumor; MN, metanephrine; N/A, not available; NMN, normetanephrine; PA, pituitary adenoma; PC, pheochromocytoma; PGL, paraganglioma; VT, vagal tumor; SDHAF2, SDH complex assembly factor 2.

^*a*^ *SDHAF2* variants were detected after molecular analysis of retrospectively reviewed patients previously diagnosed with PGLs/PCs.

^*b*^Penetrance when only at-risk family members (paternal transmission) are considered.

^*c*^Gender of patients who developed PGLs/PCs.

^*d*^The lesion located in the head of the pancreas was considered to be either a metastasis or a primary PGL.

^*e*^Of the 43 patients from the different families of whom the study of catecholamines and metanephrines was carried out.

### Treatment and Follow-up Period

Throughout follow-up (Table 1), metastatic disease was observed in 2 patients (6.1%). One patient with nonsecreting multifocal head and neck PGLs showed affected cervical and para-aortic lymph nodes and lung and bone metastases (dorsal and lumbar spine, sacrum, scapula, and costal arches) 24 years after the diagnosis of the PGLs. The only patient previously described without head and neck PGLs and a single mediastinal PGL showed bone metastases (dorsal lumbar spine and ilium) at the time of diagnosis. He died at the age of 63 years due to the disease, 3 years after the diagnosis. Regarding the concomitant development of other tumors, 3 patients were diagnosed with esophageal, cervical, and endometrial cancer during follow-up. Another patient developed an ovarian endometrioma and another 2 subjects lipomas.

As for the treatment of PGLs/PCs (Table 1), 28 (84.8%) patients have undergone or are currently awaiting surgery. Radiosurgery was selected as the therapy of choice in 3 subjects because of the location of their head and neck PGLs; 8 patients received concomitant radiotherapy. All of these subjects except 1 presented with multifocal tumors. The patient with a single mediastinal PGL and metastatic disease, apart from surgery and radiotherapy, also received treatment with a tyrosine kinase inhibitor (sunitinib). In 2 patients, active surveillance was decided upon taking into account their basal state or young age. A total of 15 (45.5%) patients experienced complications derived from the treatment and during follow-up, the most common being vocal cord paralysis (7 [21.2%]) and cranial nerve palsy (6 [18.2%]).

### Imaging Study With [68Ga]Ga-DOTA-TOC and [18F]DOPA PET/CT

**Figure 2.**
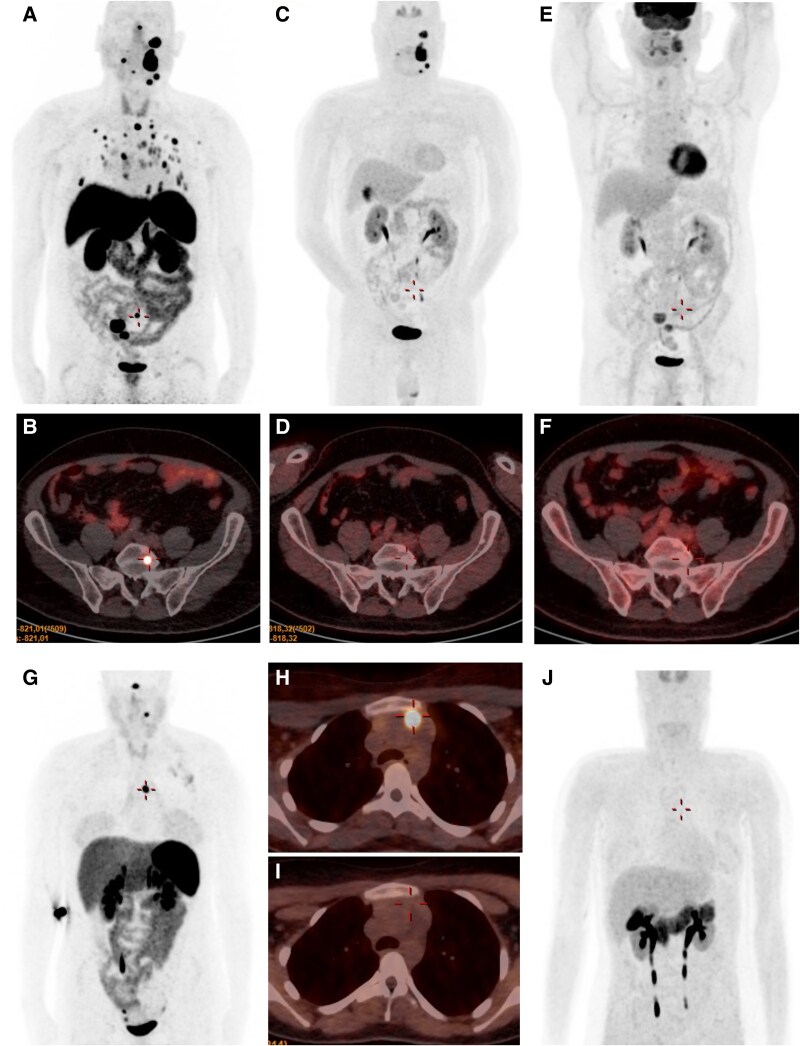
Comparative functional images of patients with extracervical and malignant paragangliomas associated to the *SDHAF2* gene. A 54-year-old man with multifocal head and neck paragangliomas along with bone and lung metastases according to [68Ga]Ga-DOTA-TOC PET/CT (A, B). [18F]DOPA PET/CT (C, D) and ^18^F-FDG PET/CT (E, F) failed to show focal uptake of the metastatic lesions. A 14-year-old woman with intense focal [68Ga]Ga-DOTA-TOC uptake at the head and neck (G) and mediastinal level (G, H) and negative [18F]DOPA PET/CT (I, J).

A comparative imaging study with both [68Ga]Ga-DOTA-TOC and [18F]DOPA PET/CT was carried out in 22 subjects with PGLs (Table 3, Fig. 2). Anatomical imaging with MRI or CT was used as the reference standard for the evaluation of the detection rates of both PET/CT modalities. They showed a total of 98 tumors and metastatic lesions, of which 93 were detected by [68Ga]Ga-DOTA-TOC PET/CT, whereas a total of 48 were missed by [18F]DOPA PET/CT. Overall detection rate on a per-patient basis was 95.7% (95% CI, 91.3-100; *P* = .125 in comparison with CT/MRI) for [68Ga]Ga-DOTA-TOC PET/CT and 79.3% (95% CI, 66.0-92.6; *P* = .004 in comparison with CT/MRI) for [18F]DOPA PET/CT. Although [68Ga]Ga-DOTA-TOC PET/CT was able to verify all head and neck PGLs seen on CT/MRI, it failed to detect 1 extracervical PGL and 4 metastatic lesions. However, 2 PGLs seen with this technique were not detected by anatomical imaging. As for [18F]DOPA PET/CT, a total of 9 head and neck PGLs and 4 extracervical PGLs were missed. The greatest handicap for [18F]DOPA PET/CT was the evaluation of metastatic disease; thus, it was only able to visualize 3 of 38 metastatic lesions (Table 3, Fig. 2).

**Table 3.** Number of paragangliomas and metastases detected according to imaging modality and anatomical distribution in a subset of patients with *SDHAF2*-related familial paraganglioma syndrome

Type of lesion	[68Ga]Ga-DOTA-TOC (n lesions)	[18F]DOPA (n lesions)	CT/MRI (n lesions)	Detection rate [68Ga]Ga-DOTA-TOC (%)*^a^*	Detection rate [18F]DOPA (%)*^a^*
All PGLs and metastatic lesions	93	50	98	95.7	79.3
All PGLs	59	47	60	97.2	85.2
Head and neck PGLs	50	41	50	100	84.5
Extracervical PGLs	9	6	10	91.7	68.7
Metastases	34	3	38	93.9	4.0

[68Ga]Ga-DOTA-TOC and [18F]DOPA PET/CT scans were carried out on a subset of patients (n = 22) with *SDHAF2*-related tumors. Combined cross-sectional imaging with computed tomography or magnetic resonance imaging was used as the reference standard for the evaluation of the detection rates of functional imaging modalities.

Abbreviations: CT, computed tomography; MRI, magnetic resonance imaging; PET, positron emission tomography; PGL, paraganglioma.

^*a*^Detection rate of [68Ga]Ga-DOTA-TOC and [18F]DOPA PET/CT on a per-patient basis.

On the other hand, the SUV_max_ data of the tumors with the highest uptake were evaluated for both PET/CT modalities (Table 4). Although mean SUV_max_ of all lesions was 65.8 ± 62.5 g/mL for [68Ga]Ga-DOTA-TOC PET/CT, a lower uptake was observed for [18F]DOPA PET/CT (15.1 ± 12.6 g/mL), *P* = .001. Similar results can be observed when comparing PGLs within the different localizations between both imaging techniques. Although a tendency toward a lower uptake for the extracervical tumors in comparison with the head and neck PGLs was observed, no statistically significant differences were found.

**Table 4.** Analysis of mean SUV_max_ of the paragangliomas and metastases with the highest uptake with [68Ga]Ga-DOTA-TOC and [18F]DOPA PET/CT in a subset of patients with *SDHAF2*-related familial paraganglioma syndrome

Type of lesion	[68Ga]Ga-DOTA-TOC SUV_max_	[18F]DOPA SUV_max_	*P* value
All PGLs and metastatic lesions (g/mL)	65.8 ± 62.5	15.1 ± 12.6	.001
All PGLs (g/mL)	67.8 ± 63.4	15.5 ± 12.7	.001
Head and neck PGLs (g/mL)	79.9 ± 69.2	18.6 ± 12.9	.002
Extracervical PGLs (g/mL)	36.3 ± 30.7	5.6 ± 4.1	.017
Metastases (g/mL)	43.3 ± 35.8	3.9 ± 0.8	.0001

[68Ga]Ga-DOTA-TOC and [18F]DOPA PET/CT scans were carried out on a subset of patients (n = 22) with *SDHAF2*-related tumors. Data are expressed as mean ± SD.

Abbreviations: CT, computed tomography; PET, positron emission tomography; PGLs, paragangliomas; SUV_max_, maximum standardized uptake value.

### Histopathological Study

**Figure 3.**
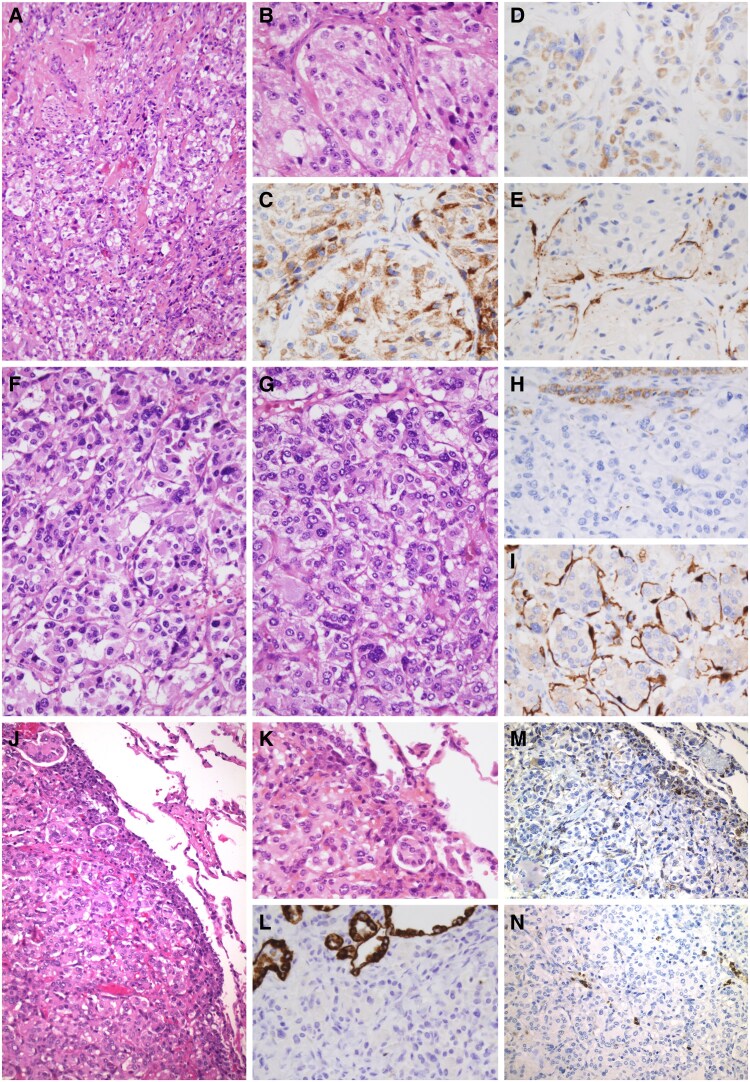
*SDHAF2*-related familial paraganglioma and phaeochromocytoma. Mediastinal paraganglioma (A and B, hematoxylin-eosin) showing strong cytoplasmic positivity for chromogranin A (C) and SDHB (D), as well as for S100 in the sustentacular cells (E). Right-sided (F) and left-sided (G) pheochromocytoma from the same patient. Both tumors showed SDHB negativity (positive internal control in cortical cells, upper portion of the image) (H), and well-formed nests of tumor cells highlighted by S100-stained sustentacular cells (I). Pulmonary metastasis of paraganglioma (J and K), showing negativity for cytokeratins (clone AE1/AE3) (L), variable cytoplasmic positivity for SDHB (M) and lack of sustentacular cells positive for S100 (N). Original magnification: A, J, M, and N, 200×; B-I, K, and L, 400×.

A subset of PGLs/PCs (n = 13) from patients with the p.(Gly78Arg) variant in the *SDHAF2* gene that underwent surgery was exhaustively evaluated to determine their microscopic features. The main clinicopathological and immunohistochemical characteristics of the study are shown in Table 5 and Fig. 3. The arrangement of the PGL/PC cells in islets (zellballen pattern), delimited by S100-positive sustentacular cells, was evident in the histopathological study in most tumors (11 [84.6%]), whereas only 2 tumors demonstrated areas of large nest formation or a pseudorosette pattern. Capsular invasion was identified in 10 (76.9%) tumors and vascular invasion in only 1 tumor. Extension into the adjacent adipose tissue was observed in 2 cases. All tumors were highly cellular except for 1, which was interpreted to have low cellularity. Monotonous cells were identified in 3 PGLs and tumor cell spindling in 2 PGLs (1 malignant). Mitotic figures were inconspicuous or absent in the majority of tumors, with only 1 benign tumor showing atypical mitotic forms. Nuclear pleomorphism was noted in 4 tumors and nuclear hyperchromasia in 7 tumors. Necrosis was noted in only 1 benign tumor. Ki-67 was variable in expression, with 53.8% of cases showing <1% of the tumor nuclei reactivity. In the 2 patients with malignant PGLs, Ki-67 was found to be 1% to 3% and >3% in the tumors evaluated and a Ki-67 > 3% was also observed in 2 benign tumors. Mean GAPP score in all tumors was 3.5 ± 1.4, with 84.6% of PGLs/PCs being suggestive of a moderate-differentiated type (including the malignant cases). On the other hand, mean PASS (including both PGLs and PCs) was 4.7 ± 1.9, with 69.9% of tumors presenting a score ≥4. The immunohistochemical study of SDHB in the tumor tissue was shown to be negative in 11 tumors and positive in 2 tumors. Additional somatic genetic analysis of these 2 PGLs showed the conservation of the wild type allele (no loss of heterozygosity).

**Table 5.** Microscopic features of a subset of paragangliomas and phaeochromocytomas

Microscopic feature/score	No. of tumors with feature present (n = 13)
GAPP score	
0-2, n (%)	2 (15.4)
3-6, n (%)	11 (84.6)
7-10, n (%)	0 (0.0)
PASS	
<4	4 (30.7)
≥4	9 (69.2)
Histological pattern	
Zellballen, n (%)	11 (84.6)
Large and irregular cell nest, n (%)	1 (7.7)
Pseudorosette (even focal), n (%)	1 (7.7)
Central or confluent necrosis, n (%)	1 (7.7)
Cellularity	
Low (<150 cells/U), n (%)	1 (7.7)
Moderate (150-250 cells/U), n (%)	0 (0.0)
High (>250 cells/U), n (%)	12 (92.3)
Cellular monotony, n (%)	3 (23.1)
Tumor cell spindling, n (%)	2 (15.4)
Mitotic figures	
>3/10 HPF, n (%)	0 (0.0)
Atypical forms, n (%)	1 (7.7)
Nuclear pleomorphism, n (%)	4 (30.7)
Nuclear hyperchromasia, n (%)	7 (53.8)
Capsular invasion, n (%)	10 (76.9)
Vascular invasion, n (%)	1 (7.7)
Extension into adipose tissue, n (%)	2 (15.4)
Ki-67	
<1%, n (%)	7 (53.8)
1-3%, n (%)	3 (23.1)
>3%, n (%)	3 (23.1)
Positive SDHB, n (%)	2 (15.4)
S100 sustentacular cell,s n (%)	13 (100)

A GAPP score of 0-2 is suggestive of a well-differentiated type; a score of 3-6 is suggestive of a moderate-differentiated type; a score of 7-10 is suggestive of a poorly differentiated type. A PASS score <4 is suggestive of tumors that behave in a benign fashion; a score ≥4 suggests a potential for a biological aggressive behavior.

Abbreviations: GAPP, Grading System of Adrenal Phaeochromocytoma and Paraganglioma; HPF, high-power field; PASS, Pheochromocytoma of the Adrenal Gland Scaled Score.

## Discussion

Although the involvement of the SDHx genes family in the development of PGLs/PCs is now better understood as a clinical complex, the clinical characterization of *SDHAF2*-related familial paraganglioma syndrome in particular still remains largely elusive. The present dataset, encompassing a substantial number of subjects with this syndrome, casts light upon several gaps present in the existing literature and offers crucial insights into its genotype-phenotype correlation. In summary, although there is a predominant development of nonsecreting multifocal head and neck PGLs at an early age, this syndrome can occur with extracervical PGLs and, less frequently, with PCs. Metastatic disease can also be observed in tumors that microscopically seem to have potentially malignant behavior. Increased normetanephrine and 3-methoxytyramine may be detected within secreting PGLs/PCs, and the combination of anatomical and molecular imaging with [68Ga]Ga-DOTA-TOC PET/CT should be considered, if available, to fully delineate the extent of the disease.

The first pathogenic variant in the *SDHAF2* gene associated to the presence of head and neck PGLs was described in 2009 (13, 16). Since then, because of the rarity of this disorder, only individual cases or case series limited to small samples have been reported (17-24), leading to the current scarcity of knowledge about this specific syndrome. In this sense, although all other PGL syndromes with variants in the SDH complex have been more or less frequently associated with extracervical and adrenal tumors, it was initially thought that *SDHAF2* variants were only associated with head and neck PGLs (17), until 2014, when the 2 first subjects with PCs were described (21). In 2019, another case with PCs was reported (24). Two more subjects from our cohort, who were found to have PCs at presentation (1 showing bilateral tumors) have been added to this case history. In addition, a further 9 patients in our study showed mediastinal PGLs (some with cardiac involvement) and 3 patients abdominal extra-adrenal PGLs (33% of cases). As in the case of most *SDHD*-related PGLs (13) and the other *SDHAF2* families reported (13-17), this tumor development was found to be inherited paternally in all patients, also showing a highly penetrant phenotype, similar to that described for the *SDHD* gene (31). Considering that both *SDHAF2* and *SDHD* show the same paternal transmission of the phenotype and that they are both located on chromosome 11, it is likely that the inheritance explanation would be the same for both genes (25, 26).

Extracervical PGLs seem to behave more aggressively in comparison with the head and neck location (32). In general terms, it is known that up to 40% of all PGLs develop metastasis (4, 33, 34), with a reported median overall survival of 7 years (32). In addition, more than 50% of all subjects with metastatic PGLs/PCs appear to carry cluster 1 pathogenic variants (35-37) (mainly *SDHB*-related tumors, followed by *SDHD*-related tumors) (12, 36-38). However, to date, the metastatic rate of *SDHAF2*-related PGLs/PCs is still unknown. Here, metastatic disease associated with this specific gene is reported in 2 of 33 patients (<6%) presenting nonsecreting head and neck PGLs and a nonsecreting mediastinal PGL, with an overall prevalence of 4% (mainly in the lungs and bone) when the families and individual cases previously reported are also taken into account.

Although there are several parameters considered to be related with a greater metastatic potential (tumor size, location, secreting phenotype, the presence of an *SDHB* pathogenic variant, and Ki-67 index), to date, there are no reliable biological or histopathological markers for predicting metastatic dissemination (33, 35, 39). Regarding histopathology, the PASS and GAPP scores are the only risk-stratification systems available (29, 30). However, further studies are needed to elucidate if the potential metastatic behavior of these tumors can be accurately predicted with these grading systems (40). In the current *SDHAF2* cohort, the GAPP score of the PGLs/PCs evaluated was greater than 3 in 84.6% of the tumors evaluated and PASS (although originally designed for the evaluation of PCs) was greater than 4 in 69.2% of PGLs/PCs, therefore suggesting a potentially malignant behavior for these tumors. In fact, the most frequent microscopic characteristics of the tumors were the presence of high cellularity (92.3%) and capsular invasion (76.9%). Within the GAPP parameters, Ki-67 has been considered of particular interest and it is general accepted that PGLs/PCs with high Ki-67 have a greater probability of presenting a malignant course (41, 42). In contrast to the above for our cohort, more than half of the tumors evaluated had a Ki-67 < 1. However, it is also known that PGLs/PCs with very low Ki-67 sometimes show metastatic lesions. For this reason, this parameter has not been accepted as a single marker for malignant behavior. In fact, it has already been reported that cellularity may be a more important parameter than Ki-67 itself (30). On the other hand, although it is well known that immunohistochemical staining of tumor tissue for SDHB provides a valuable method for identifying patients likely to have SDHx pathogenic variants, retained expression of SDHB protein was found by immunohistochemistry in 2/13 tumors. To our knowledge, only 2 other cases of immunohistochemical positivity for SDHB in PGLs associated with *SDHB* germline variants have previously been reported (43). In our series, the reactivity of the 2 SDHB-positive tumors was confirmed by a new immunohistochemical analysis, and additional somatic genetic analysis showed the conservation of the wild-type allele (no loss of heterozygosity). Thus, although immunohistochemistry is a useful screening tool, it should be interpreted carefully for the detection of variants in the SDHx gene family.

As for tumor functionality, different secreting phenotypes have been established depending on the genotype. Thus, most PGLs/PCs belonging to cluster 1 and more specifically to the SDHx genes family, present with a noradrenergic phenotype. In addition, a dopaminergic phenotype has also been described for this gene complex, with increased levels of 3-methoxytyramine and a greater risk of malignancy (33). In this *SDHAF2* cohort, most of the patients exhibited nonsecreting PGLs, considering that less than 4% of head and neck PGLs are associated to an increased secretion of catecholamines (44). However, in accordance with this information, the 2 patients with PCs showed high normetanephrine levels and an increased secretion of 3-methoxytyramine was also observed in 1 patient with head and neck PGLs.

Another characteristic of the tumors related to the *SDHAF2* gene is multifocality, which was present in 78.8% of patients in our cohort and in 50% to 91% in the previous reports available (14, 16). In addition, young age is also a common feature in the development of these multifocal tumors (16), with an average age at onset of 38 years in our cohort, with the youngest patient being diagnosed with PGLs at the age of 12 years. In this sense, over the years, cascade screening and advances in imaging modalities have led to earlier detection of these tumors in asymptomatic patients, thereby reducing the age at diagnosis. The development of other tumors related to the underlying genetic predisposition of the SDHx genes family has also been described as gastrointestinal stroma tumor and pulmonary chondroma within Carney-Stratakis syndrome, renal cell carcinoma, pituitary adenoma, and neuroblastoma in the case of *SDHB* (8). As is the case of our cohort, the presence of other tumors has also been described (18), which, given their low prevalence among family members and the rarity of *SDHAF2*-related PGLs/PCs, they are assumed to be unrelated incidental findings.

Regarding imaging modalities, CT provides excellent anatomical detail and high sensitivity for the screening of PGLs/PCs, but low specificity (45). Although MRI was shown to be superior to CT in the screening of extra-adrenal PGLs, it can also give worse spatial resolution and greater motion artifacts (4, 46). As for the cluster 1 PGLs/PCs in particular, the highest sensitivity and specificity for their detection is provided by molecular imaging (32, 47). In addition, PGLs/PCs within the SDHx genes family in general highly express the somatostatin receptor 2, which is why [68Ga]Ga-DOTA-TOC PET/CT is considered to be the most sensitive imaging technique in the screening and diagnosis of these tumors (4, 10, 47). *SDHAF2*-related tumors showed to be no different from the rest of the SDHx genes family and [68Ga]Ga-DOTA-TOC PET/CT proved to be superior to [18F]DOPA PET/CT in the detection of multifocal PGLs and metastatic lesions in the current study. The mean SUV_max_ of [68Ga]Ga-DOTA-TOC was also higher than that of [18F]DOPA in both PGLs and metastatic lesions. The different expression of norepinephrine transporters and somatostatin receptors of these tumors could explain this situation. On the other hand, although [68Ga]Ga-DOTA-TOC PET/CT failed to detect 1 extracervical PGL, 2 PGLs detected with this technique were not seen by anatomical imaging, suggesting that the combination of anatomical and molecular imaging is needed to completely delineate the extent of the disease (48).

Taking into account the evident positivity of these tumors with [68Ga]Ga-DOTA-TOC PET/CT, which demonstrates the expression of somatostatin receptors 2, valuable information can be obtained with this technique for the use of peptide receptor radionuclide therapy in patients with metastatic disease (49). In addition, therapy with “cold” somatostatin analogues may be expected to achieve similar results to those already found for other neuroendocrine tumors, as shown in the clinical trials carried out by the PROMID and CLARINET study groups (50, 51). However, although lanreotide is currently being studied in metastatic PGLs/PCs in a phase II clinical trial (ClinicalTrials.gov Identifier: NCT03946527), to date, literature regarding the use of these drugs in this population is still scarce (52) and prospective studies are urgently needed.

This study presents the largest series with *SDHAF2*-related familial paraganglioma syndrome reported to date, in which the clinical, biochemical, histological, and comparative radionuclide imaging evaluation of PGLs/PCs specifically associated with this gene is comprehensively shown. However, it also has some limitations. A haplotype analysis using four SNPs in the *SDHAF2* gene among the index cases of each family presented here did not exclude a link between the families. Indeed, such a link is likely if the high frequency of the variant for a health care area of 450 000 people is also taken into account. In the case of the 2 Spanish and Dutch families previously described with the same variant, there was no clear evidence of relatedness, suggesting that p.(Gly78Arg) is probably a recurrent, rather than a founder, variant and that this residue is relevant to the function of *SDHAF2* (13, 16, 17). On the other hand, as shown in Fig. 1, molecular analysis was not available for all the family members of each pedigree and, therefore, the frequency and penetrance of this specific genetic change could vary slightly.

In conclusion, the present dataset contributes to augmenting the knowledge of the clinical characterization of *SDHAF2*-related familial paraganglioma syndrome, showing the presence of nonsecreting multifocal head and neck and extracervical PGLs at an early age and, less frequently, PCs. Increased normetanephrine and 3-methoxytyramine levels can be observed within secreting PGLs/PCs and metastatic dissemination can also be detected in tumors that microscopically seem to show potentially malignant behavior. Molecular imaging with [68Ga]Ga-DOTA-TOC PET/CT should be considered in order to obtain valuable information about tumor extent and possible therapies. Universal genotyping including the *SDHAF2* gene in the early detection of cervical and extracervical PGLs/PCs has a valuable role in the management of patients with this syndrome.

## Data Availability

Restrictions apply to the availability of some or all data generated or analyzed during this study to preserve patient confidentiality. The corresponding authors will on request detail the restrictions and any conditions under which access to some data may be provided.

## Abbreviations

CT: computed tomography
GAPP: Grading System of Adrenal Phaeochromocytoma and Paraganglioma
MRI: magnetic resonance imaging
PASS: Pheochromocytoma of the Adrenal Gland Scaled Score
PC: pheochromocytoma
PET: positron emission tomography
PGL: paraganglioma
SDH: succinate dehydrogenase
SDHAF2: SDH complex assembly factor 2
SUV_max_: maximum standardized uptake value
